# Nursing Institutions and Hospitals

**Published:** 1887-02-26

**Authors:** J. C. Steele, Bedford Fenwick, G. W. Potter


### NURSING INSTITUTIONS AND HOSPITALS.

By Dr. J. C. Steele, Mr. Burdett, Dr. Bedford Fenwick, and Dr. G. W. Potter.

There was a very full attendance at the Hospitals Association Meeting on Wednesday, the 16th inst., to hear Miss Manson's paper, published in last week's Hospital, page 345. Amongst those present were:

Mrs. Field, late Matron of Guy's Hospital; Miss Medhill, Matron of St. Mary's Hospital; Mrs. Suckling, Lady Superintendent of the Royal Hants County Hospital; Miss Smith, Miller Memorial Hospital; Miss Cooper, Victoria Hospital for Children; Miss Robertson, Lady Superintendent, St. Helena Home; Miss D. M. Stockah, Hull Nursing Home; Miss Mollett, National Hospital for the Paralysed; Miss Franks, Kensington Infirmary; Mrs. Burdett; Miss Louisa Smith, Cottage Hospital, Blackheath; Miss Davies, East London Hospital, Shadwell; Miss Ferry, Poplar Hospital; Miss Fowler; Miss Lee, Bristol; and many other ladies. Colonel Montefiore, and Messrs. P. Michelli, Secretary of St. Mary's; T. A. Hind, Home Hospitals Association; H. Collins, Lock Hospital; Edward Clifford, A. Field, Dr. E. Percival Cockey, Keith, D. Young, and H. Hall.

The following discussion took place.

Dr. G. W. Potter, who presided, expressed the hope that they might be favoured with some observations by the ladies. As there were so many ladies present, it would be very profitable and instructive, and certainly in accordance with custom, if some of them would take part in the discussion. He cordially invited them to remain seated, but to speak.

No lady responding,

Dr. Steele (Superintendent of Guy's Hospital) observed that as no lady could be persuaded to rise, he supposed he must break the ice, but he would do so only for the purpose of expressing the thanks of the meeting to Miss Manson for her able paper. Miss Manson seemed to put the heading of her paper in the form of a question, and she had answered that question in the affirmative, so far as concerned the advisability of the hospitals possessing nursing institutions from which the public could be supplied with nurses. All connected with the large hospitals would come to the same conclusion, because, during the past ten or fifteen years in the development in the nursing of our public hospitals, all had felt the necessity of enlarging the number of nurses attached to the hospitals. They had found it an absolute necessity to extend the numbers, in consequence of the tremendous call for extra nurses. He calculated in a hospital having a hundred beds with a hundred nurses, only seventy might be found continually occupied in staff nursing. He estimated ten per cent. would be sick; ten per cent. away for their holidays; and ten per cent. more engaged with special nursing; so that would leave no more than seventy for staff nursing. Nursing institutions had in a great measure become a necessity for hospitals to supplement the regular staff of nurses. At Guy's they had now twelve nurses on special duty, and Mrs. Wardroper told him she had twenty thus engaged. Work formerly done by students, such, for example, as that connected with tracheotomy and ovariotomy cases, was now delegated to nurses, and he thought very properly so. The charges at nursing institutions were very reasonable, and he believed that it would result in second-class nurses being driven out of the field altogether. Poor people and the lower middle classes could not afford high charges, and it was very difficult to get good parish nurses to attend them. He had been applied to on several occasions to select good parish nurses, and he found great difficulty. He expected the remuneration was not so good as it ought to be. He desired to express his thanks to Miss Manson for her very useful and suggestive paper.

No lady could be induced to speak, and so, after an interval,

Mr. Burdett said he must reluctantly give the remarks then which he had intended to reserve till later in the evening. He regarded this question as presenting three important aspects, *i.e.*, the educational, economic, and public.

The Educational Aspects.

We were all of us largely dependent upon education for our views, and our views largely governed our actions Those who were old enough, and had had the privilege of belonging to the workers in our hospitals during the past twenty-five years, knew that the improvements which had been made in nursing had been a matter of education. He said a matter of education, because it was once thought that hospitals, like workhouses, were institutions where everything must be done cheaply. This cheap policy had been fatal to efficiency, and for many years made it largely difficult, if not wholly impossible, while it made nursing an apology for what it ought to have been. In 1865 it was thought that anybody did for a night nurse; poor women, who did charing and washing all day, were generally paid ten shillings a week as night nurses, with the privilege of sleeping in the hospital wards, instead of in their beds at home. Such an aspect of the question would strike his hearers as being as ridiculous, as it was cruel in its effects upon the suffering and the sick in the hospital wards, but in those days it was thought quite an adequate system. Night nurses at this period were often chosen because they were found to be the least capable. The medical profession were at first rather sceptical about the advantages of trained nurses, because they felt that the ladies who took up trained nursing might be drawn from those who aspired to be lady doctors, and that they would gradually attempt to assimilate to themselves the functions of the doctor as opposed to those of the nurse. Nursing institutions were better without any woman, whatever her qualifications as a nurse, who aspired to become a lady doctor, because he was confident that a nurse, however capable she was, must always be the handmaiden to the doctor, and not his rival in the hospital ward. As soon as this fact came to be recognised and insisted upon, it placed nursing in a higher position than it ever occupied before. He ventured to think, speaking to his present audience, that they did not wish to arrogate to themselves the functions of the doctor, and that they regarded nursing as a high and a noble profession to which it was an honour to belong. So much for the educational view.

The Economical Aspects.

Turning from the educational to the economic aspect of the question, Mr. Burdett pointed out that the value of Miss Manson's paper was due to the fact that it brought out the economic value of nursing institutions. There had been a discussion in that room about a month ago on the subject of the Metropolitan Poor-law Infirmaries, and it was then said that it was true that the nursing in some of those establishments was not so efficient as it might be, though in others it was very good. The average guardian declared that the ratepayers would not submit to the expenses of proper nursing, but good nursing would really be the cheapest He looked forward to the time when we should have training schools for nurses attached to every institution which treated sickness, so that the wants of the public might be adequately met in the supply of nurses, a supply which was lamentably deficient at the present time. He ventured to think that, in another twenty years, trained nursing would not be regarded as an extravagance. He believed that if to enable the hospitals to start nursing institutions some more funds were necessary in the way of capital, they would be abundantly forthcoming from the profits made by (*a*) nursing institutions, and (*b*) the awakened interest of the public when they found that they could always send a servant to the hospital and get a thoroughly reliable trained nurse for their children, or perhaps for themselves.

The Public Aspect.

There was a great deal of humanity in man, and you could touch him closely and deeply by proving to him that by giving a contribution to the hospital, he was paying something which would return him a hundredfold when his own turn came to be overtaken by illness or accident. We might all some day be laid low in this way, and so find ourselves dependent on others. Any woman who was engaged in nursing was engaged in a noble calling—a calling which every man and woman amongst us, whatever their occupation, ought to recognise and be grateful for. All thoughtful persons must thank God for the fact that we had an increasing army of women who devoted themselves to this work, not only in the hospitals and in private families, but also in district-nursing in the homes of the poor, by whose agency an amount of human suffering was alleviated which it was impossible to estimate, and which would have its reward hereafter. He was most anxious that we should adopt a system of sifting the chaff from the wheat in respect to nurses. If we could do that we should have done a very great justice to legitimate nursing and a very great service to the public.

Pensions for Nurses.

He hoped that one other aspect of this question might be advanced in this year of Jubilee. He referred to the recognition of the fact, that if a woman devoted her life to nursing, when she was too old to nurse the sick and had herself to be nursed and cared for, a provision ought to be found for her. Lady Bloomfield recently came to him and expressed her willingness, subject to the consent of her committee and existing liabilities, to make the money she had collected the neuclus for a National Pension Fund for nurses of all grades. The nurse ought to be free from any fear of destitution and loss of independence when she grew old, because she should know there was a provision on which she could live till her life's end. Another aspect of this pension fund was that it should be provident, and a nurse leaving her profession for marriage or another sphere of duty, should be allowed to have her money back. Although the movement had been delayed because of the difficulty of awakening the hospital committees to a sense of its need, still we were now measurably nearer to the successful establishment of a National Pension Fund than ever before. The scheme, which had been drawn by a leading actuary, was all ready to be put in force, and only awaited the co-operation of those, whose interests were involved, to make it a great boon to many workers amongst the sick and a genuine success. He was sure they were all very much indebted to Miss Manson for her paper. Miss Manson was the head of one of the largest and oldest of our hospitals, and no words of his were necessary to justify her selection as the reader of the paper they were discussing.

Dr. Bedford Fenwick said that Miss Manson was well known for two very remarkable characteristics. First, an apparently intuitive perception of the salient points of a question however difficult. Secondly, of the power of expressing her ideas in the fewest words, and in the clearest possible language. That power had been exemplified in the paper they had just heard. The whole subject of hospitals had been thoroughly threshed out; first, the relation of hospitals to the public and to the patients, and to the medical and nursing professions; then the question of private nursing. Although the paper extended only over two small sheets, Miss Manson had really gone into the very minutest details. As Mr. Burdett had said, some few years ago it used to be taken for granted that anybody might be a nurse, and it was so, to a certain extent, even now. It was very important that nurses should be supplied by institutions which could guarantee their efficiency. Some nursing institutions took not the slightest trouble to find out what good trained nurses were; they simply farmed out nurses. Speaking of the treatment of nurses by the public, he observed that the question was a most important one. The nurse did an immense amount of work; she did it ungrudgingly; her work was hard, and sometimes very ungratefully received. The way in which nurses were treated at most nursing institutions was unfair both to the public and to the nursing profession. He gave examples of this which had come under his own notice, while at the same time fully admitting that some nursing institutions did their work conscientiously and well. Nursing, he considered, was *the* profession for women. It made men respect the truest and purest instincts of womanhood, and did more in this way than anybody could well realise. It had effects that were far-reaching; it showed an example of pure good work—the most important work that a woman could do. He often wished Sir Walter Scott had known the modern nurse, for then he felt sure he would have modified those sarcastic lines in *Marmion:*

> "O woman! in our hours of ease,
> Uncertain, coy, and hard to please,
> And variable as the shade
> By the light quivering aspen made;
> When pain and anguish wring the brow,
> A ministering angel thou!"

After a few remarks from Mr. A. O. D. Bartholeyns, Secretary of the Middlesex Hospital,

The Chairman said the discussion had been very interesting, but he felt it his duty to scold the ladies present for not having spoken. (Laughter.) Dr. Steele had brought out a very important point which Miss Manson (purposely, no doubt) had overlooked—the lack of trained nurses for the lower middle and poorer classes. Mankind was one, and, in any scheme for establishing nursing institutions at the great hospitals, the lower middle and poorer classes must not be forgotten. The Hospitals Association, he might mention, intended to have a Register of Nurses kept at the offices of the Association, and were about to publish the rules under which all certificated nurses might enter their names thereon. The object the Council had at heart in establishing this Register of Nurses was to enable the public to know for certain whether a nurse was certificated and trained or not. Each candidate who applied and was allowed to become registered would have a badge, which she could show to the world as proof that she was a fully qualified and registered nurse. Dr. Bedford Fenwick had analysed Miss Manson's paper so well that he could not wish it done better. The main point of the discussion was, of course, this: that nurses should be trained by general hospitals which could guarantee their efficiency, and that the public should be enabled to obtain them from those institutions. There were nursing institutions now existing where really excellent nurses could be obtained. From one (the Mildmay Park Institution) he had had a great many nurses, and had only come across one who was inefficient. He wished to say that, in view of the somewhat indiscriminate charges made against existing nursing institutions. He thought the meeting was greatly indebted to Miss Manson for her excellent paper; it was one of the best they had had. With regard to lady doctors, he did not agree with Mr. Burdett. Miss Manson, for instance, if she could write a paper like that, could make a very good doctor if she chose. The nurse was not only the doctor's right hand—she was his *alter ego*—his other self. Nothing gave a doctor so much real help as a good nurse. He himself could not possibly speak too highly of nurses, and he hoped that large and efficient nursing institutions might be established at all hospitals. He proposed that the best thanks of the meeting be given to Miss Manson for her admirable paper.

Mr. Burdett, in proposing a vote of thanks to Dr. Potter for taking the chair, and another to Dr. Bedford Fenwick for his kindness in reading the paper, said that he had no objection to lady doctors. He once said to Mrs. Garrett Anderson, when she was Miss Garrett, "If you wish to be a lady doctor, and if all other ladies in the country desire to do the same, by all means persevere; only don't try to upset the management of existing medical institutions by entering their schools. Have a school of your own, and I will be very happy to subscribe to it." What he urged was, that when a lady took up nursing as a profession, she should keep to it, and not seek to become a doctor through a nursing home. In America he was informed that the lady doctors found it so hard to get a living that they were deserting the profession and taking to nursing.

Dr. Steele, in seconding the votes to the chairman and the reader of the paper, said they were much indebted to Dr. Potter for presiding and for his remarks.

Dr. Potter having briefly replied on behalf of himself and Dr. Bedford Fenwick, the proceedings terminated.

